# ADAR1 and MicroRNA; A Hidden Crosstalk in Cancer

**DOI:** 10.3390/ijms18040799

**Published:** 2017-04-11

**Authors:** Charles J. Cho, Seung-Jae Myung, Suhwan Chang

**Affiliations:** 1 Department of Biomedical Sciences, University of Ulsan College of Medicine, Asan Medical Center, Seoul 05505, Korea; formidable1981@gmail.com; 2 Department of Gastroenterology and Convergence Medicine, University of Ulsan College of Medicine, Asan Medical Center, Seoul 05505, Korea; sjmyung@amc.seoul.kr

**Keywords:** ADAR (Adenosine deaminase, RNA specific), UTR (untranslated region), NGS (Next Generation Sequencing)

## Abstract

The evolution of cancer cells is believed to be dependent on genetic or epigenetic alterations. However, this concept has recently been challenged by another mode of nucleotide alteration, RNA editing, which is frequently up-regulated in cancer. RNA editing is a biochemical process in which either Adenosine or Cytosine is deaminated by a group of RNA editing enzymes including ADAR (Adenosine deaminase; RNA specific) or APOBEC3B (Apolipoprotein B mRNA Editing Enzyme Catalytic Subunit 3B). The result of RNA editing is usually adenosine to inosine (A-to-I) or cytidine to uridine (C-to-U) transition, which can affect protein coding, RNA stability, splicing and microRNA-target interactions. The functional impact of these alterations is largely unclear and is a subject of extensive research. In the present review, we will specifically focus on the influence of ADARs on carcinogenesis via the regulation of microRNA processing and functioning. This follows a brief review of the current knowledge of properties of ADAR enzyme, RNA editing, and microRNA processing.

## 1. The Role of RNA Editing in Eukaryotes

Well-known post-transcriptional modifications of eukaryotes include 5′-capping, 3′ poly-adenylation, alternative splicing, and RNA editing, as has been highlighted by recent studies [1,2]. RNA editing is a process that modifies nucleotide sequences of RNA transcripts without altering its template genomic DNA [3]. RNA editing aids in providing diversity of transcriptome, and possibly proteome from limited source of gene set [2]. A number of RNA editing events has been reported in vertebrates to date. Among these, the best characterized type of RNA editing event is adenosine to inosine (A-to-I) conversion, which is mediated by the ADAR (Adenosine deaminase; RNA specific) family of enzymes [2,4]. In most eukaryotic processes, Inosine is recognized as Guanosine. Therefore, A-to-I editing of RNA can alter the genetic codon to introduce different amino acid, produce or delete alternative splicing site, and subsequently influence mRNA and protein level. 

## 2. ADAR and A-to-I Editing

### 2.1. Target Sites of A-to-I Editing and ADAR Binding

Initial discoveries of A-to-I editing events were rarely, and serendipitously made when comparing cDNA sequences with their counterpart genomic DNA in coding regions. Several editing events in protein coding regions were found to alter protein levels and thus affect change in cell function. Thanks to recent advance in sequencing technology (i.e., Next-generation sequencing), several databases are available to predict editing of RNAs using NGS (Next Generation Sequencing) data (RADAR: http://rnaedit.com, DARNED: http://darned.ucc.ie) [5,6,7,8]. Recent studies have revealed that the vast majority of human A-to-I editing sites are located in non-coding regions, especially inversely oriented *Alu* elements [5,9,10]. Thus, it can be postulated that ADAR induced A-to-I editing may play a role in biogenesis of microRNAs (miRNAs), which are short, noncoding RNAs that post-transcriptionally regulate gene expression, or editing of their targets (e.g., seed sequence of 3′ UTR). In addition, a recent investigation using CLIP (Cross Link Immuno-Precipitation)-Seq method reported that about 15% of binding sites were found to be located in non-*Alu* regions, which provides functional roles related to regulation of alternative 3′ untranslated region (UTR) usage and primary miRNA processing in the nucleus of ADAR [11].

### 2.2. Structure, Expression, and Localization of ADARs

**Figure 1:**
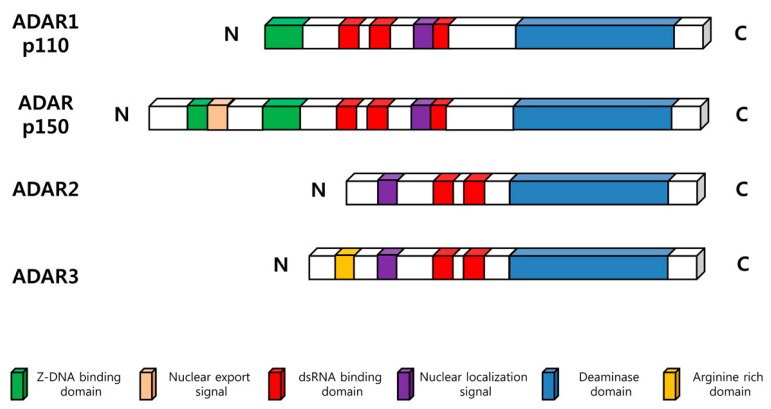
A schematic diagram showing functional domains of Adenosine deaminase; RNA specific (ADAR) protein family. Two splicing isoforms of ADAR1, p110 and p150, are shown on top. The other two ADARs, ADAR2 and ADAR3 are shown below. Each colored box indicates conserved functional motifs.

**Figure 2:**
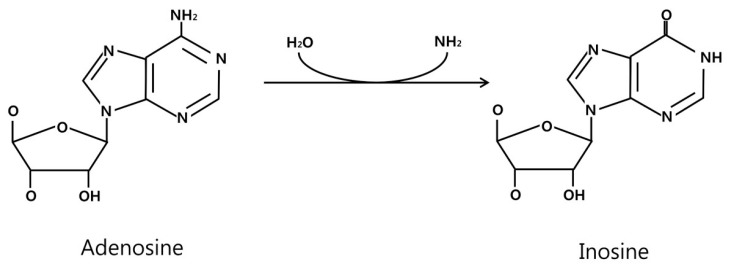
A chemical reaction catalyzed by ADAR. The amine group in the Adenosine on the left is removed by ADAR that produces Inosine. The inosine is recognized as Guanine, thus generating A to G conversion.

There are three members of known ADAR family of enzymes (ADAR1, ADAR2, and ADAR3) in vertebrates (Figure 1). All ADARs share two common functional domains. Deaminase domain located in the carboxy-terminal region plays a central role in hydrolytic deamination at the C6 position (Figure 2). Analysis of crystal structure of the catalytic domain of ADAR2 revealed that inositol hexaphosphate (IP6), which is buried within the enzymatic core, contributes to protein folding [12]. In addition, double stranded RNA-binding domain (dsRBD), which has an α-β-β-β-α configuration, enables ADARs to make contact to dsRNA, providing substrate selectivity of the enzyme [13]. For binding of ADAR, dsRNA length and base pairing of dsRNAs seem to play a pivotal role [4]. Indeed, dsRNA modifying activity required a dsRNA of at least 15–20 base pairs for substrate recognition and modification efficiency below 100 bp was found to be relatively low [14]. It has been reported that both ADAR1 and ADAR2 have 5′ neighbor preference (A=U > C=G), and ADAR2 may have 3′ neighbor preference (U=G > C=A) [15]. In addition, presence of snoRNA (e.g., MBII-52) has also been shown to decrease the efficiency of ADAR2 function [16]. The specific characteristics of each ADAR enzyme are shown as follows.

Regarding the genetically engineered mouse (GEM) model of ADAR, the ADAR1 knockout mice are known to be embryonic lethal [17,18], and ADAR2 knockout mice showed postnatal death due to A-to-I editing of *GRIA2* gene. However, the ADAR2 KO lethality can be rescued by the introduction of homozygous *GRIA2* Q/R knock-in allele [19]. On the other hand, the effect of ADAR1 overexpression in mouse was studied in B-cells using Lck promoter-driven transgenic mouse [20]. For ADAR2, a transgenic model has been generated and showed behavior disorders due to the serotonin (5HT) 2C receptor (5HT(2C)R) misediting [21,22].

### 2.3. ADAR1

ADAR1 is encoded by *ADAR* gene, located on chromosome 1q21, and is ubiquitously expressed in mammals. The gene encodes proteins of two sizes with separate promoters; p110 isoform (~110 kDa) and p150 isoform (~150 kDa) [23]. It is well known that the former isoform is constitutively active and the latter can be induced in interferon producing conditions [24]. In addition to the editing function of ADAR, the role of p150 isoform in suppressing interferon response has been widely studied. ADAR1 mutant mice die by embryonic day E12.5 with overproduction of interferon [18]. Mutations of *ADAR* gene cause Aicardi-Goutières syndrome, which is characterized by childhood encephalopathy and massive interferon production [25]. In addition, the deadly phenotype of ADAR1 knockdown mouse has been shown to be reversible with concomitant knockdown of MAVS (Mitochondrial antiviral signaling protein) or MDA5 (Melanoma Differentiation-Associated protein 5) [26,27]. It has been recently demonstrated that A-to-I editing of endogenous dsRNA by ADAR1 is essential to prevent the activation of the cytosolic dsRNA response by endogenous transcripts [26]. Relationship of ADAR with a variety of viruses has also been reported. ADAR1 is demonstrated to be proviral, antiapoptotic host factor with regard to measles virus infection [28]. However, ADAR1 was shown to inhibit vesicular stomatitis virus growth in response to interferon treatment [29].

In addition to three dsRBD domains as well as the deaminase domain in the C-terminus, both ADAR1 isoforms contain Zβ domain, a Z-DNA binding site. However, only p150 isoform contains the Zα domain, which contains a nuclear export signal (NES) [30]. Subcellular localization of both ADAR1 isoforms includes nucleus and cytoplasm. The nuclear localization signal (NLS) located in the third dsRBD drives the nuclear import of both isoforms of ADAR1. In contrast, nuclear export of p150 isoform is mediated by binding of exportin 1 to the NES, the mechanism of which is different from the export of p110 isoform, requiring exportin 5 [31]. 

### 2.4. ADAR2

ADAR2 is encoded by *ADARB1* gene, located on chromosome 1q21 [32]. Although ADAR2 is ubiquitously expressed, it is known to be mostly expressed in the brain. The gene encodes 741 amino acids and 80.8 kDa sized protein. ADAR2 N-terminus contains a NLS, which allows ADAR2 to localize into the cell nuclei [33]. ADAR2 is known to edit critical position of the AMPA (α-amino-3-hydroxy-5-methyl-4-isoxazolepropionic acid) receptor subunit GluR-B pre-mRNA in the brain, which results in the conversion of glutamine residue to an arginine [34]. ADAR2 knockdown mice die young and are prone to seizures. Their phenotype is reversible when an unedited transcript is substituted with an edited one [19].

### 2.5. ADAR3 

ADAR3 is encoded by the *ADARB2* gene, located on chromosome 10p15, and is known to be expressed at detectable levels only in the brain [35,36]. The gene encodes 739 amino acids and 80.6 kDa sized protein. ADAR3 carries the Arginine-rich domain (R domain), which has been demonstrated to bind to both single stranded- and double stranded-RNA, in addition to two dsRBDs [36,37]. Although catalytic residues of the ADAR family members are also present in ADAR3, its deaminase activity has not been demonstrated to date [36]. Therefore, this enzyme will not be discussed in the present review. 

## 3. Overwhelming Evidence of RNA Editing by ADARs in Cancer

**Figure 3:**
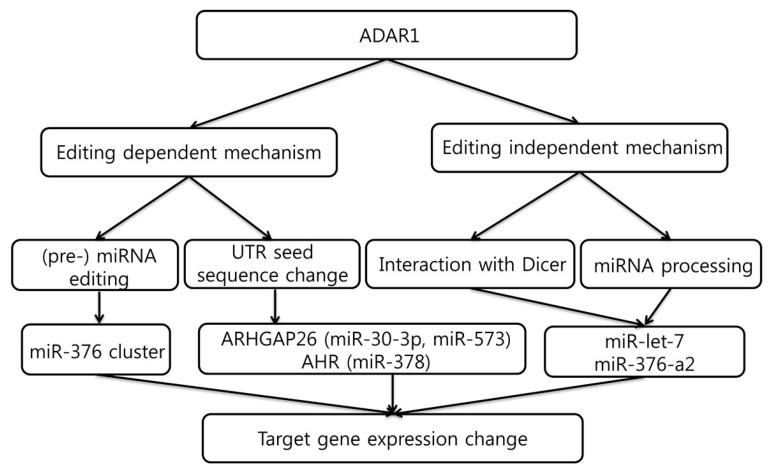
Hypothetical classification for the mechanisms of ADAR1-mediated miRNA editing and gene expression change. As described in the text, there is a deaminase activity-dependent editing of miRNA seed sequence or target gene untranslated region (UTR) (on **left**) whereas the deaminase activity independent mechanism involves ADAR1 interaction with Dicer or effect on the Drosha processing (on **right**). Both mechanisms merge into the change of target gene expression eventually.

Cancer is increasingly recognized as a constellation of heterogeneous clones, the development of which follows the Mendelian trait [38]. This heterogeneity leads to sub-clonal selection within the tumor ecosystem, which potentially gives us the chance to understand its invasive and metastatic potential and ability to evade therapy. Although genetic mutations of genes play a key role in driving tumorigenesis, RNA editing by ADARs that provide additional diversity in various tumor types has recently begun to be unveiled. The functional effect of ADAR is exerted via two mechanisms; editing-dependent or editing-independent. The former pathway encompasses editing of microRNA or nucleotide change of its seed sequence in 3′-UTR. The latter pathway is related to microRNA processing in the nucleus or ADAR’s direct interaction with Dicer in the cytoplasm (Figure 3).

Recently, Paz-Yaacov et al. performed a transcriptome-wide analysis of RNA editing events in the coding region using the cancer genome atlas (TCGA) data, and showed that elevated events of A-to-I editing and level of ADAR1 in most cancer types, which also was found to be associated with patient survival [39]. The authors suggested that a few of the edited RNAs serve as drivers that might be novel candidates for therapeutic and diagnostic purposes. Notably, RNA sequencing data derived editing events in the UTR regions were also found, which showed coinciding editing candidates across various cancer types. However, since RNA sequencing data cannot discriminate between genetic mutation and RNA editing events without genomic DNA sequencing data, comparison of data between whole genome sequencing and RNA sequencing should be performed. Indeed, the authors obtained whole-genome sequencing data from International Cancer Genome Consortium and whole-exome sequencing data from TCGA, and excluded RNA editing candidates when potential mutational signals were found.

In addition, Han et al. also underwent bioinformatic assay to investigate RNA editing profiles across 17 cancer types [40]. The authors used RADAR database as a reference to identify candidates of RNA editing. Notably, over-editing cancer types were a predominant feature, although under-editing cancer type was also present (e.g., kidney chromophobe). Several RNA editing events in the coding sequence were found, including *AZIN1* (Antizyme Inhibitor 1) at S367G, *COPA* (Coatomer subunit alpha) at I164V, *COG3* (Conserved oligomeric Golgi complex subunit 3) at I635V, and *GRIA* (glutamate ionotropic receptor AMPA type subunit 1) at R764G. Interestingly, among the RNA editing events in the coding sequence, *AZIN1* S367G, *COG3* I635V, and *GRIA* R764G significantly increased cell viability. In addition, alteration in the response to cancer therapeutics was found: *AZIN1* S367G for IGF-1R inhibitor, *GRIA* R764G for MEK inhibitor and *COG3* I635V for MEK inhibitor and trametinib.

Chan et al. reported that ADAR1 over-expression and ADAR2 down-regulation in tumors demonstrated an increased risk of liver cirrhosis and postoperative recurrence as well as poorer prognosis in hepatocellular carcinoma [41]. The authors also demonstrated that ADAR1 functions as an oncogene while ADAR2 acts as a tumor suppressor in vitro and in vivo assays.

In gastric cancer, a similar role of ADAR1 and ADAR2 as oncogene and tumor suppressor was reported [42]. Using an exemplary target gene *PODXL* (podocalyxin-like), the authors demonstrated that the ADAR2-regulated recoding editing at codon 241 (His to Arg) confers a loss-of-function phenotype that neutralizes the tumorigenic ability of the unedited *PODXL*. Qin et al. reported the overexpression of ADAR1 in primary esophageal cancer cell line due to gene amplification [43]. The phenomenon leads to hyperediting of *FLNB* (Filamin B) and *AZIN1* mRNA, the latter candidate shows a gain-of-function phenotype, leading to aggressive tumor behavior [43]. However, Nemlich et al. reported that in situ analysis of metastatic melanoma show substantial downregulation of ADAR1 [44]. These results corroborate the idea that the expression and functional role of ADARs are organ- and cancer type-specific.

## 4. MicroRNA Biogenesis and Influence of RNA Editing 

MicroRNAs are small RNAs of ~22 nucleotides can target mRNAs and thereby function as post-transcriptional regulators [45]. Mature miRNAs are initially transcribed as several thousand nucleotides long primary microRNA (pri-miRNA) in the nucleus [45]. Subsequently, pri-miRNA folds to form hairpin structures, known as precursor-microRNAs (pre-miRNAs) of up to 70 nucleotides in length. The pre-miRNA is generated by 650 kDa-sized RNase III protein Drosha, in complex with the pri-miRNA recognition factor DiGeorge syndrome Critical Region gene 8 (DGCR8) [46]. Pre-mRNA duplexes are exported to the cytoplasm, where they are processed further by RNase III protein, Dicer, in complex with TAR RNA-binding protein (TRBP) to generate double-stranded, mature miRNA duplex of ~22 nt in length. Mature miRNAs are then loaded onto Argonaute proteins (Ago) 1–4, to form the core of the RNA-induced silencing complex (RISC) [47]. Interestingly, previous reports showed that ADAR1 can edit microRNAs [48], which will be described in detail below.

## 5. Editing-Dependent ADAR Effects in Cancer

The easiest postulation on how RNA editing affects tumorigenesis would be through the editing of nucleotide sequencing in the coding region, microRNA binding site or miRNA itself. The alteration of miRNA binding site is of interest since the majority of the editing sites are in non-coding regions. Comparing the sequence of transcriptome before and after knock-down in a cancer cell line may provide firm evidence on the role of ADAR1 in RNA editing, since knockdown of the enzyme will directly decrease the frequency of editing. We have recently knocked down ADAR with lentiviral transfection of shRNA, and compared the frequency of editing events before and after the experiment. Most of the editing events were A-to-I, and occurred in the 3′ UTR region, which decreased upon ADAR1 knockdown [49]. 

Likewise, a number of previous reports have demonstrated editing-dependent ADAR effects either on miRNA itself or its binding target sequence. Choudhury et al. found that RNA editing of miR-376 cluster, which is induced by ADAR2, is reduced in human gliomas, with accumulation of the unedited form of miR-376a-3p [50]. The unedited miRNA promotes glioma cell migration and invasion, whilst the edited form inhibits these capacities in vitro. 

On the other hand, Wang et al. provided another mechanism of ADAR1 activity to regulate miRNA-mediated modulation of UTR site of target gene and its expression [51]. A-to-I RNA editing events were found within the 3′ UTR of *ARHGAP26* (Rho GTPase activating protein 26), encoding the Rho GTPase activating protein 26. The authors revealed that both miR-30b-3p and miR-573 can target unedited ARHGAP26 UTR, which in edited form becomes resistant to repression by these two miRNAs. In addition, Nakano et al. recently reported another evidence for the editing of target UTR in hepatocellular carcinoma [52]. Creation of a novel miR-378 recognition site in the *AHR* 3′-UTR was observed after ADAR1 knockdown increased the aryl hydrocarbon receptor (AhR) protein levels and induced the downstream target gene *CYP1A1* (Cytochrome P450, family 1, subfamily A), without affecting *AHR* mRNA level. Thus, the authors suggested that the RNA-editing-dependent down-regulation of AhR by miR-378 contributes to the variability in the constitutive hepatic expression of AhR. Interestingly, despite large differences in ADAR1 expression in human liver samples, the inter-individual differences in the RNA editing levels within the *AHR* 3′-UTR were relatively small, suggesting that there may be a threshold of ADAR level to induce sufficient editing rate.

## 6. Editing-Independent Effects of ADAR

In addition to A-to-I sequence changes on miRNAs or their targets, the evidences of ADAR function via an editing-independent mechanism was also revealed. Nemlich et al. reported that ADAR1 regulates Dicer expression via let-7 in metastatic melanoma. In addition, they reported that ADAR1 formed a complex with DGCR8 that was mutually exclusive with the DGCR8-Drosha complex that processes pri-miRNAs in the nucleus [44]. Heale et al. reported that both catalytically active and inactive ADAR2 can modulate the processing of mir-376a2 by disrupting Drosha processing, thereby emphasizing the role of ADAR enzymes as RNA-binding proteins apart from their RNA editing activity [53]. Ota et al. found that ADAR1 forms a complex with Dicer through direct protein-protein interaction [54]. This Dicer/ADAR1 heterodimer complex reportedly increases the maximum rate of pre-miRNA cleavage and facilitates miRNA loading onto the RISC complex, thereby promoting the silencing of the target gene. Consistent with these reports, we also found the knockdown of ADAR1 in gastric cancer cell (MKN-45) led to altered miRNA expression (Table 1, unpublished data). Fifty-one miRNAs showed decreased expression level by more than 50% and 17 miRNAs had increased expression level after ADAR1 knockdown, which corroborates that ADAR1 promotes miRNA processing. Editing of pre-miRNA or miRNA occurred relatively infrequently, with only one case of editing being found in the seed sequence.

## 7. MicroRNAs That Regulate ADAR1

**Figure 4:**
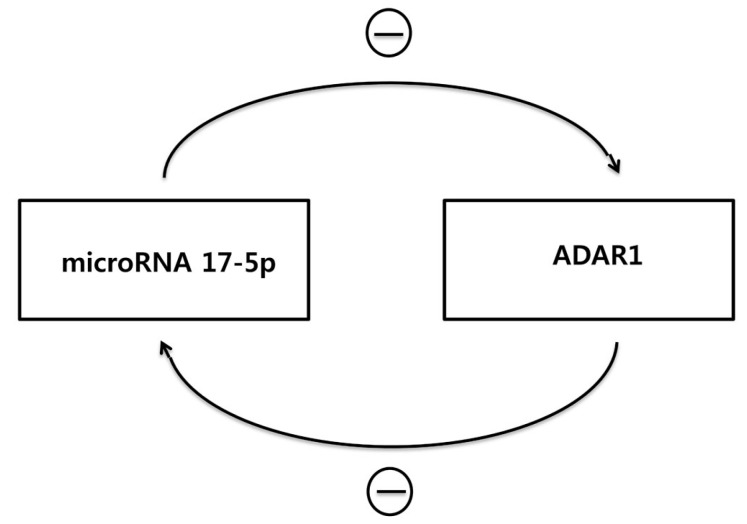
Overexpression of ADAR1 forms a feedback loop with miR-17-5p. Based on the previous reports and our unpublished data, it could be postulated that the overexpression of ADAR1 forms a feedback loop starting with a decreased miR-17-5p level, that in turn up-regulates ADAR1. ⊝ indicates negative regulation.

Apart from ADAR’s role in altering miRNA function and/or its level, it could also be postulated that ADAR expression is regulated by miRNA. Indeed, Lim et al. demonstrated that miR-1 interacts with 3′ UTR of ADAR1 and subsequently decreases the mRNA expression of ADAR1 [55]. In addition, miR-17-5p and mi-432-5p, which are frequently overexpressed in melanoma, were reported to silence ADAR1 mRNA in melanoma cell line [44]. Although obtained from a cell line derived from different types of tumor (stomach cancer derived MKN-45 cell line), knock-down of ADAR1 increased miR-17-5p level by 47.9% in our experiment. In this regard, it could be postulated that overexpression of ADAR1 forms a feedback loop with decreased miR-17-5p, which in turn up-regulate ADAR1. (Figure 4)

## 8. Conclusions

One of the unique and important features of the RNA editing is transientness. As most of the RNA have a short lifetime, the RNA editing event by ADAR1 can be robust but does not last as long as the edited RNA decays. Considering the cancer cells face many challenges, including immune cell attack, nutrient limitation, hypoxia as well as therapeutic agents, the RNA editing can be a useful tool for the cancer to overcome these abrupt challenges. Among the editing targets, microRNA is relatively less studied but a promising target to understand the editing-mediated cancer cell plasticity. Further studies in this area will reveal the list of miRNAs edited by RNA editing in certain conditions and address how the miRNA editing changes target gene regulations. Moreover, more research will be needed to understand how the editing-independent mechanism cooperates to change the level of specific miRNAs and its target genes. 

## Figures and Tables

**Table 1:** Number of miRNAs whose expression was changed by ADAR1 knockdown, in gastric cancer cell MKN-45. Interestingly, there are more miRNAs with the expression changes but have no differences in editing, suggesting that the editing-independent mechanism dominates in this cell.

Change in MicroRNA Expression and Editing Level upon ADAR1 Knockdown	No.
microRNA expression level increased >50%	17
microRNA expression level decreased >50%	51
Editing of pre-microRNA	4
Editing of microRNA seed sequence	1
